# Helium alters the cytoskeleton and decreases permeability in endothelial cells cultured *in vitro* through a pathway involving Caveolin-1

**DOI:** 10.1038/s41598-018-23030-0

**Published:** 2018-03-19

**Authors:** Kirsten F. Smit, Moritz Konkel, Raphaela Kerindongo, Maximilian A. Landau, Coert J. Zuurbier, Markus W. Hollmann, Benedikt Preckel, Rienk Nieuwland, Martin Albrecht, Nina C. Weber

**Affiliations:** 1 Department of Anaesthesiology, Laboratory of Experimental Intensive Care and Anaesthesiology (L.E.I.C.A), Meibergdreef 9, 1100 DD Amsterdam, The Netherlands; 2 Department of Anaesthesiology, UKSH, Campus Kiel, Kiel, Germany; 3 Laboratory of Experimental Clinical Chemistry, and Vesicle Observation Centre, Meibergdreef 9, 1100 DD Amsterdam, The Netherlands

## Abstract

Caveolins are involved in anaesthetic-induced cardioprotection. Actin filaments are located in close connection to Caveolins in the plasma membrane. We hypothesised that helium might affect the cytoskeleton and induce secretion of Caveolin. HCAEC, HUVEC and Cav-1 siRNA transfected HUVEC were exposed for 20 minutes to either helium (5% CO_2_, 25% O_2_, 70% He) or control gas (5% CO_2_, 25% O_2_, 70% N_2_). Cells and supernatants were collected for infrared Western blot analysis, immunofluorescence staining, nanoparticle tracking analysis and permeability measurements. Helium treatment increased the cortical localisation of F-actin fibers in HUVEC. After 6 hours, helium decreased cellular Caveolin-1 (Cav-1) levels and increased Cav-1 levels in the supernatant. Cell permeability was decreased 6 and 12 hours after helium treatment, and increased levels of Vascular Endothelial - Cadherin (VE-Cadherin) and Connexin 43 (Cx43) were observed. Transfection with Cav-1 siRNA abolished the effects of helium treatment on VE-Cadherin, Cx43 levels and permeability. Supernatant obtained after helium treatment reduced cellular permeability in remote HUVEC, indicating that increased levels of Cav-1 are responsible for the observed alterations. These findings suggest that Cav-1 is secreted after helium exposure *in vitro*, altering the cytoskeleton and increasing VE-Cadherin and Cx43 expression resulting in decreased permeability in HUVEC.

## Introduction

The non-anaesthetic noble gas helium induces both pre- and postconditioning *in vivo* and *in vitro*, protecting against subsequent prolonged ischaemia of an organ. Helium protected against post-ischaemic endothelial dysfunction in healthy volunteers after forearm ischaemia^1^. Due to its low-density helium is already used in patients with respiratory diseases^2^. Helium has gained interest in organ protective strategies because it does not have anaesthetic, nor hemodynamic “side effects” and could therefore be applied in numerous clinical situations of ischaemia and reperfusion^3^.

Several mediators behind helium conditioning were described, radical oxygen scavengers, the mitochondrial adenosine triphosphate regulated potassium channel (K_ATP_)^4^, and the calcium sensitive potassium channel play a role in helium induced protection^5,6^. These mediators are all located on the mitochondrial membrane, probably indicating that the key mechanism behind helium conditioning congregates on this level. Mitochondrial function is modulated by Caveolins^7,8^, which are structural proteins that play an essential role in the formation of cholesterol- and sphingolipid-enriched invaginations of the plasma membrane. These “Caveolae” contain a scaffolding domain that anchors and regulates proteins, influencing cellular processes such as signal transduction and transport^9,10^. Caveolae are known as signalosomes, and are involved in protection by conditioning^11,12^. Helium conditioning in mice resulted in a decreased level of Caveolin-1 (Cav-1) and -3 in the heart, with a concomitant increase of Cav-1 and -3 levels in the blood, suggesting that circulating factors in the blood are involved in helium induced organ protection^13^.

The endothelium plays an important role in clinical ischaemia/reperfusion (I/R) injury. It is the first organ to be affected by I/R and to respond to it, and dysfunctional endothelium can contribute to local and systemic sequelae^14^. I/R compromises the local barrier function of endothelium, resulting in leakage and tissue edema, which can elicit an inflammatory response.

The endothelium normally acts as a selective permeable barrier to fluids and solutes. Adhesive junctions between cells regulate permeability, and the actin cytoskeleton plays a key role in maintaining junctional integrity^15,16^. Actin filaments in endothelium control a variety of dynamic processes such as endo- and exocytosis, changing shape and cell-cell adhesion^17^. Cortical, circumferential actin filaments maintain stable junctions, while stress fibers (contractile actin bundles) weaken junctions by exerting centripetal force^18^. Vascular Endothelial - Cadherin (VE-Cadherin) is specifically responsible for assembly and regulation of endothelial cell-cell junctions by dictating expression and localisation of other junctional molecules. A decrease in VE-Cadherin is associated with barrier disruption and increased permeability^19^. Connexin 43 (Cx43) forms gap junctions between cells and facilitates cell-cell communication in endothelial cells, but also in cardiomyocytes^20^. This intercellular communication regulates vascular tone, endothelial function and preserves cardiac rhythm^21^. Cx43 is found, both in the sarcolemma and mitochondria. Ischaemic preconditioning causes Cx43 to translocate to the mitochondria, where it preserves ATP formation and contributes to radical oxygen species (ROS) formation^22^. Preconditioning also preserves sarcolemmal Cx43 activity during sustained ischaemia, leading to maintenance of cellular integrity and decreasing cellular edema^23^.

Although the exact mechanisms of helium conditioning are still unknown, increased circulating levels of Cav-1 possibly play a role in organ protection by helium. As actin filaments are located in close connection to Cav-1 in the plasma membrane, we hypothesized that helium induces secretion of Cav-1 and thereby might affect the cytoskeleton, possibly altering adherent junction protein VE-Cadherin and gap junction protein Cx43. This could ultimately result in alterations in cellular permeability.

## Results

### Helium alters cellular actin filament alignment in HUVEC and HCAEC

A recent study has shown that helium protects the human endothelium *in vivo*^1^. To get more insights into the protective effect of helium on a cellular level, the effect of helium on cellular actin filament alignment was assessed by actin staining of treated HUVEC.

**Figure 1:**
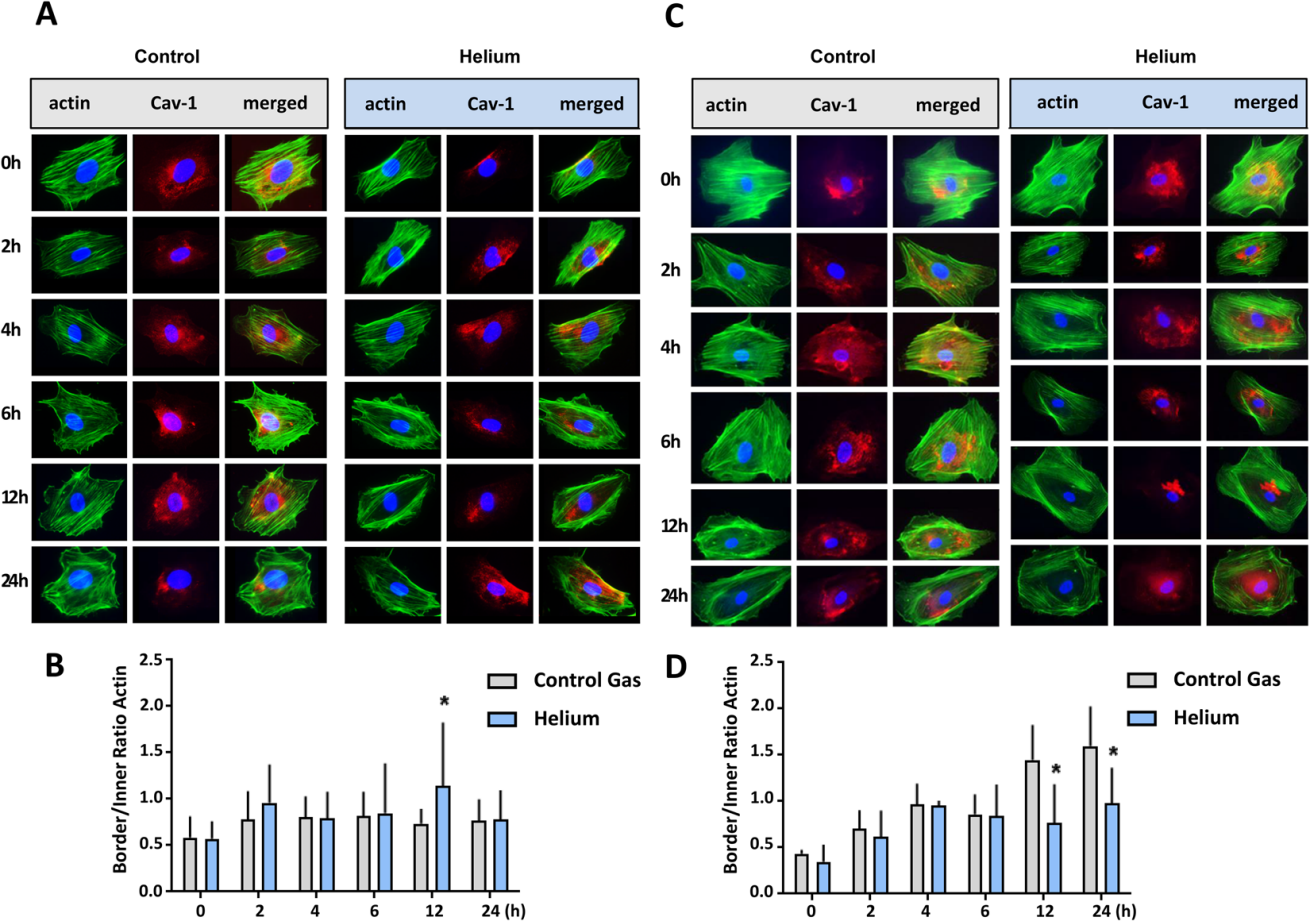
Effect of helium on cellular actin filament alignment in HUVEC and HCAEC. Panel A: Immunofluorescent staining of actin (Rhodamin), Cav-1 and nuclei (Hoechst) in HUVEC. Panel B: Graph of the inner/border ratio of actin filaments following control gas or helium treatment in HUVEC. Panel C: Immunofluorescent staining of actin (Rhodamin), Cav-1 and nuclei (Hoechst) in HCAEC. Panel D: Graph of the inner/border ratio of actin filaments following control gas or helium treatment in HCAEC. Data are represented as mean ± SD, *p < 0.05. HUVEC = human umbilical vein endothelial cells. HCAEC = human coronary artery endothelial cells.

Treatment with helium significantly increased the cortical localisation of F-actin fibers in HUVEC after 12 hours compared to cells treated with control gas, increasing the inner-to-border ratio (1.1 ± 0.7 vs 0.7 ± 0.2 respectively, shown in Fig. 1B, n = 3). After 24 hours, there was no difference observed between cells treated with helium or control gas. In HCAEC, helium treatment decreased cortical localisation of F-actin fibers compared to controls at 12 hours (0.8 ± 0.4 vs 1.4 ± 0.4 respectively) and 24 hours (1.0 ± 0.4 vs 1.6 ± 0.4 respectively, Fig. 1D, n = 4).

### Helium affects Cav-1 in HUVEC and HCAEC

Since helium leads to a translocation of Cav-1 from the myocardium to the blood in mice *in vivo*^13^, we translated these findings to the human endothelium *in vitro* and examined the effect of helium on the intra-cellular and extra-cellular Cav-1 content.

**Figure 2:**
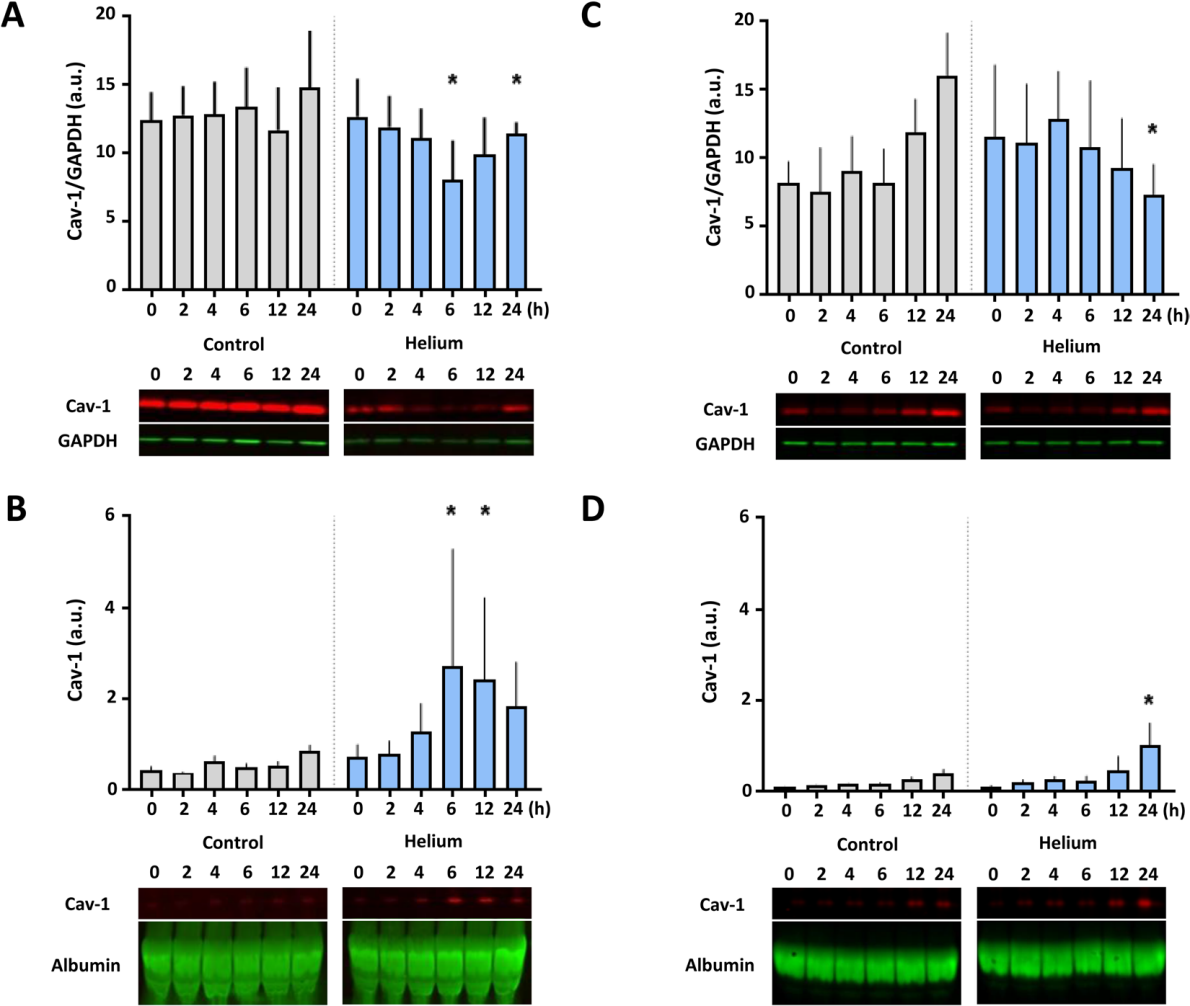
Effect of helium on Cav-1 levels in HUVEC and HCAEC. Panel A: Western blot results of the cellular ratio of Cav-1 compared to GAPDH loading controls from different time points following helium or control gas treatment in HUVEC. Panel B: Western blot results of the supernatant ratio of Cav-1 compared to albumin loading controls from different time points following helium or control gas treatment in HUVEC. Panel C: Western blot results of the cellular ratio of Cav-1 compared to GAPDH loading controls from different time points following helium or control gas treatment in HCAEC. Panel D: Western blot results of the supernatant ratio of Cav-1 compared to albumin loading controls from different time points following helium or control gas treatment in HCAEC. Cropped images of representative Western blot results are displayed below the respective graphs. Full-length blots are presented in Supplementary Figs S5–S8. Data are represented as mean ± SD, *p < 0.05. HUVEC = human umbilical vein endothelial cells. HCAEC = human coronary artery endothelial cells.

In HUVEC, helium decreases cytosolic Cav-1 in cells after 6 hours compared to controls (8.0 ± 2.9 vs 13.4 ± 2.9, p < 0.05, Fig. 2A, n = 5), and increases the level of Cav-1 in the supernatant (2.7 ± 2.6 vs 0.5 ± 0.1 p < 0.05, Fig. 2B). After 12 hours increased levels of Cav-1 are still present in the supernatant, yet the decreased level of Cav-1 in the cytosol is no longer significant. 24 hours after helium treatment, cytosolic Cav-1 is significantly decreased again compared to controls (11.4 ± 0.9 vs 14.8 ± 4.2, p < 0.05, Fig. 2A). Silver staining suggests a subtle differential release of several other proteins (25, 70 and 150 kDa) between the control and helium treated cells. However, we do not know the nature of the respective proteins yet (Figure S2, Supplementary Material).

In HCAEC, helium decreases cytosolic Cav-1 after 24 hours compared to controls (7.3 ± 2.1 vs 16.0 ± 3.0, p < 0.05, Fig. 2C, n = 6), and a concomitant increase of Cav-1 in the supernatant is found at the same time point (1.0 ± 0.4 vs 0.4 ± 0.1, p < 0.05, Fig. 2D, n = 4).

### Helium increases levels of VE-Cadherin and Cx43 in HUVEC

To reveal a possible molecular mechanism behind the helium mediated endothelial protection^1^, the effect of helium on the expression rates of the junctional molecules VE-Cadherin and Cx43 was investigated.

**Figure 3:**
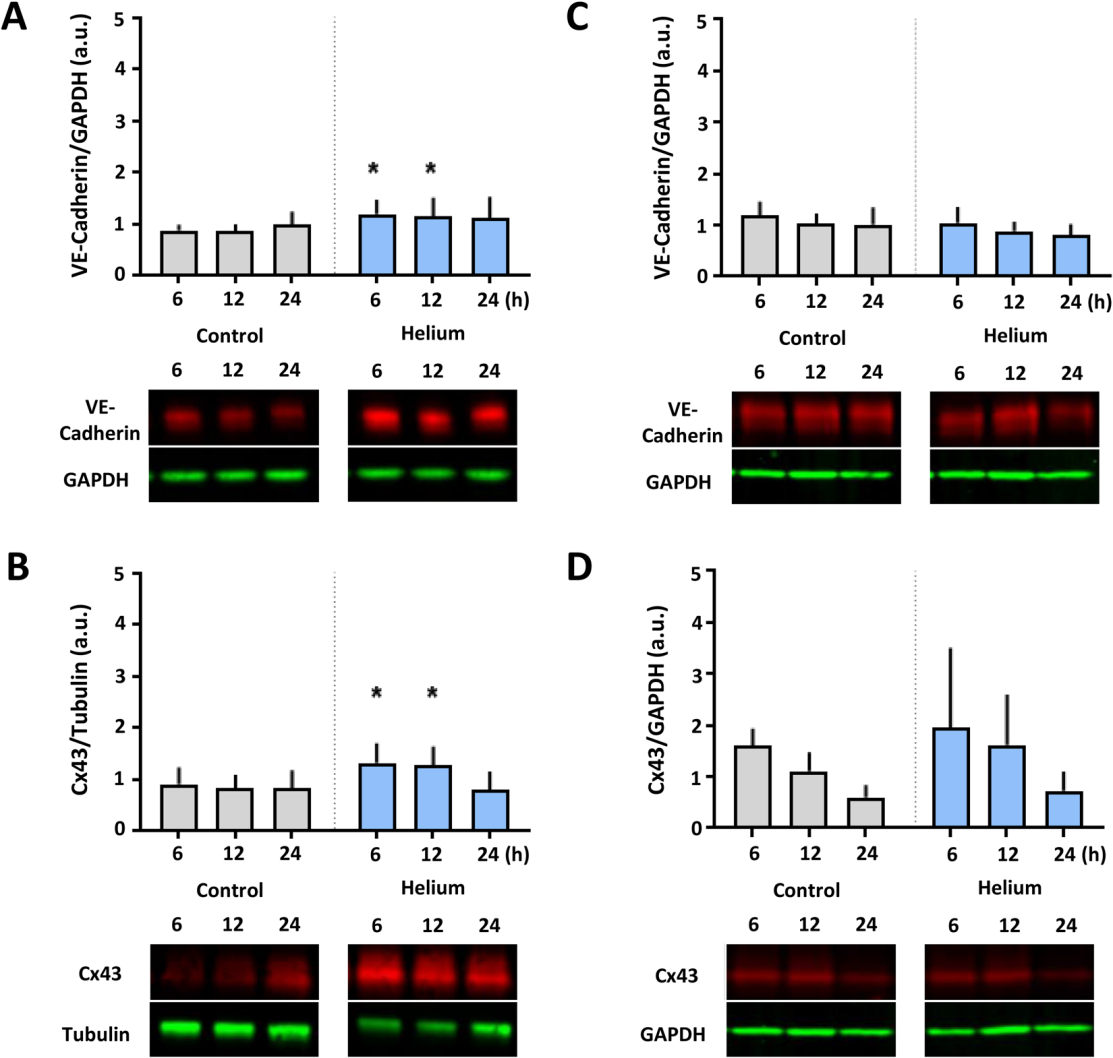
Effect of helium on VE-Cadherin and Cx43 levels in HUVEC and HCAEC. Panel A: Western blot results of the ratio VE-Cadherin compared to GAPDH loading controls in HUVEC at different time points following helium or control gas treatment. n = 14. Panel B: Western blot results of the ratio Cx43 compared to Tubulin loading controls levels in HUVEC at different time points following helium or control gas treatment. n = 11. Panel C: Western blot results of the ratio VE-Cadherin compared to GAPDH loading controls in HCAEC at different time points following helium or control gas treatment. n = 14. Panel D: Western blot results of the ratio of Cx43 compared to GAPDH loading controls in HCAEC at different time points following helium or control gas treatment. n = 14. Cropped images of representative Western blot results are displayed below the respective graphs. Full-length blots are presented in Supplementary Figs S9–S12. Data are represented as mean ± SD, *p < 0.05. HUVEC = human umbilical vein endothelial cells. HCAEC = human coronary artery endothelial cells.

Helium treatment in HUVEC increases cytosolic levels of VE-Cadherin (Fig. 3A, 1.2 ± 0.3, p < 0.05), and Cx43 (Fig. 3B; 1.3 ± 0.4, p < 0.05), after 6 hours when compared to controls (0.9 ± 0.1 and 0.9 ± 0.3, p < 0.05 respectively). This effect was still present 12 hours after helium treatment, and subsided after 24 hours. In HCAEC, levels of VE-Cadherin and Cx43 were not affected by helium (Fig. 3C,D).

### Effect of siRNA Cav-1 knock down on the effects induced by helium treatment

In order to investigate the role of Cav-1 in the helium mediated changes of the endothelial permeability and the expression rates of VE-Cadherin and Cx43, a knock-down model of Cav-1 in the human endothelium was employed.

**Figure 4:**
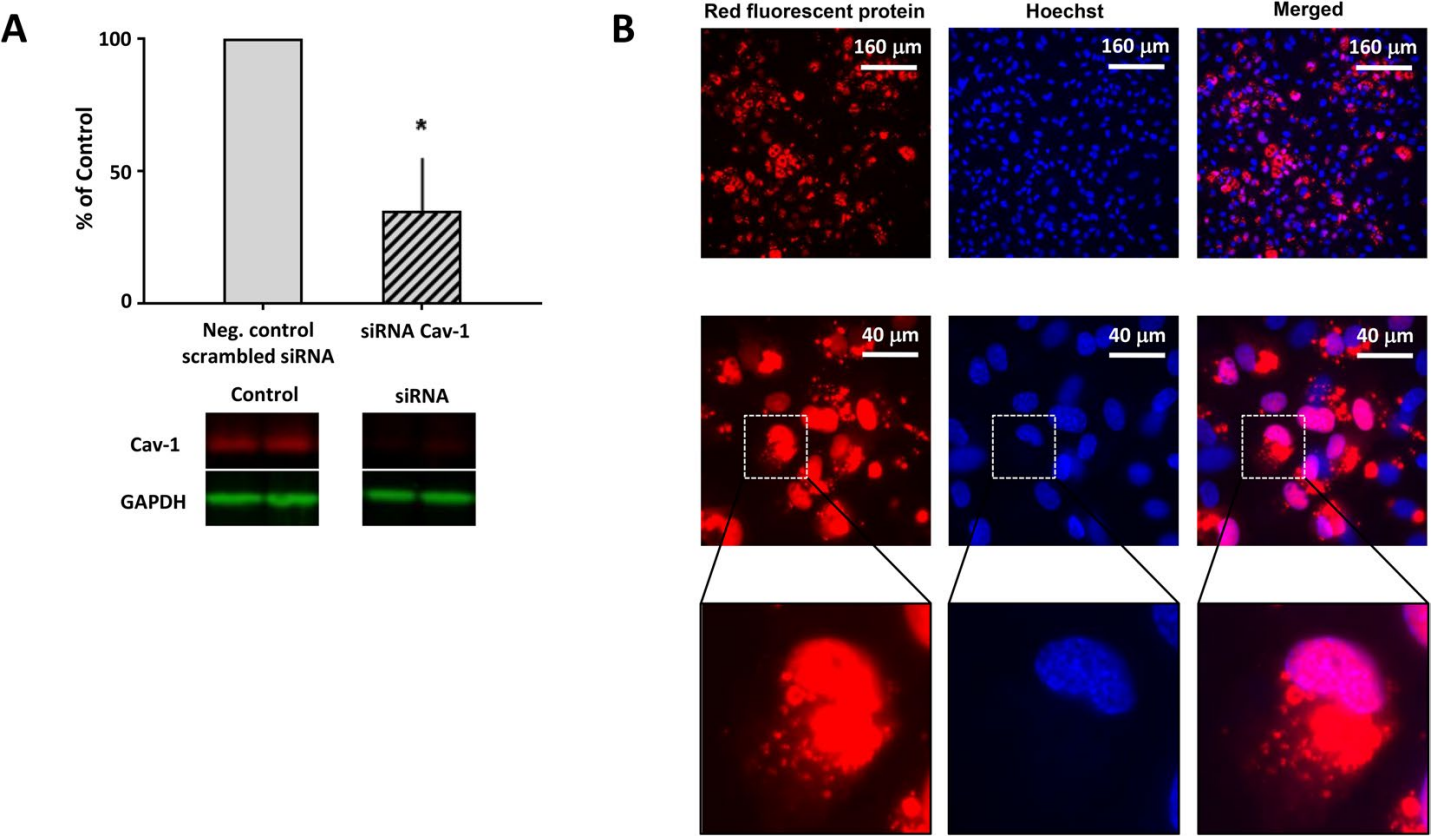
Cav-1 siRNA transfection in HUVEC. Panel A: Western blot results of the ratio of Cav-1 compared to GAPDH loading control in HUVEC transfected with siRNA for Cav-1 and negative controls. Results of the transfected cells are shown as percentage of the negative control which was set at 100%. Cropped images of representative Western blot results are displayed below the respective graph. Full-length blots are presented in Supplementary Fig. S13. Panel B: Immunofluorescent images of HUVEC transfected with a red fluorescent protein (Block IT Alexa Flour). Cells were fixed 24 hours after transfection. Images of negative control are presented in Supplementary Fig. S4.

In HUVEC, we performed a knock down of Cav-1 by transfecting the cells with siRNA for Cav-1. Transfected cells showed a significant decrease in the amount of Cav-1 compared to cells transfected with negative control scrambled siRNA (34.8 ± 20% vs 100%, p < 0.05, Fig. 4A). Figure 4B shows the immunofluorescent image of HUVEC transfected with a red fluorescent protein, indicating transfection efficiency.

### Helium influences permeability in HUVEC and this is mediated by Cav-1

**Figure 5:**
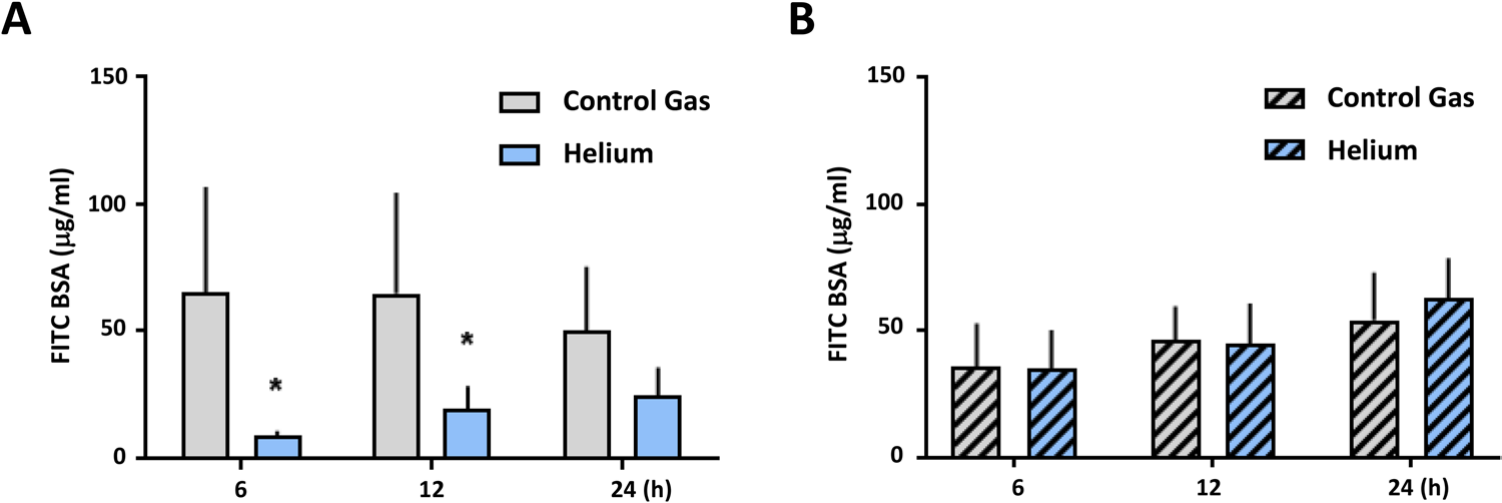
Effect of helium on permeability in HUVEC and Cav-1 siRNA transfected HUVEC. Panel A: Results of helium on permeability of a confluent monolayer of HUVEC, estimated by the transfer of FITC-BSA, at different time points compared to control gas. n = 7. Panel B: Results of helium on permeability of a confluent monolayer of Cav-1 siRNA transfected HUVEC, estimated by the transfer of FITC-BSA, at different time points compared to control gas. n = 8. Data are represented as mean ± SD, *p < 0.05. HUVEC = human umbilical vein endothelial cells.

In HUVEC, helium treatment decreases cellular permeability after 6 hours compared to controls (Fig. 5A, 9.0 ± 1.7 vs 65.2 ± 41, p < 0.05), and this effect remained present after 12 hours (19.5 ± 8.8 vs 64.8 ± 39, p < 0.05), but not after 24 hours (Fig. 5A). In HCAEC, helium does not significantly alter permeability at any time point (Figure S3, Supplementary Material).

**Figure 6:**
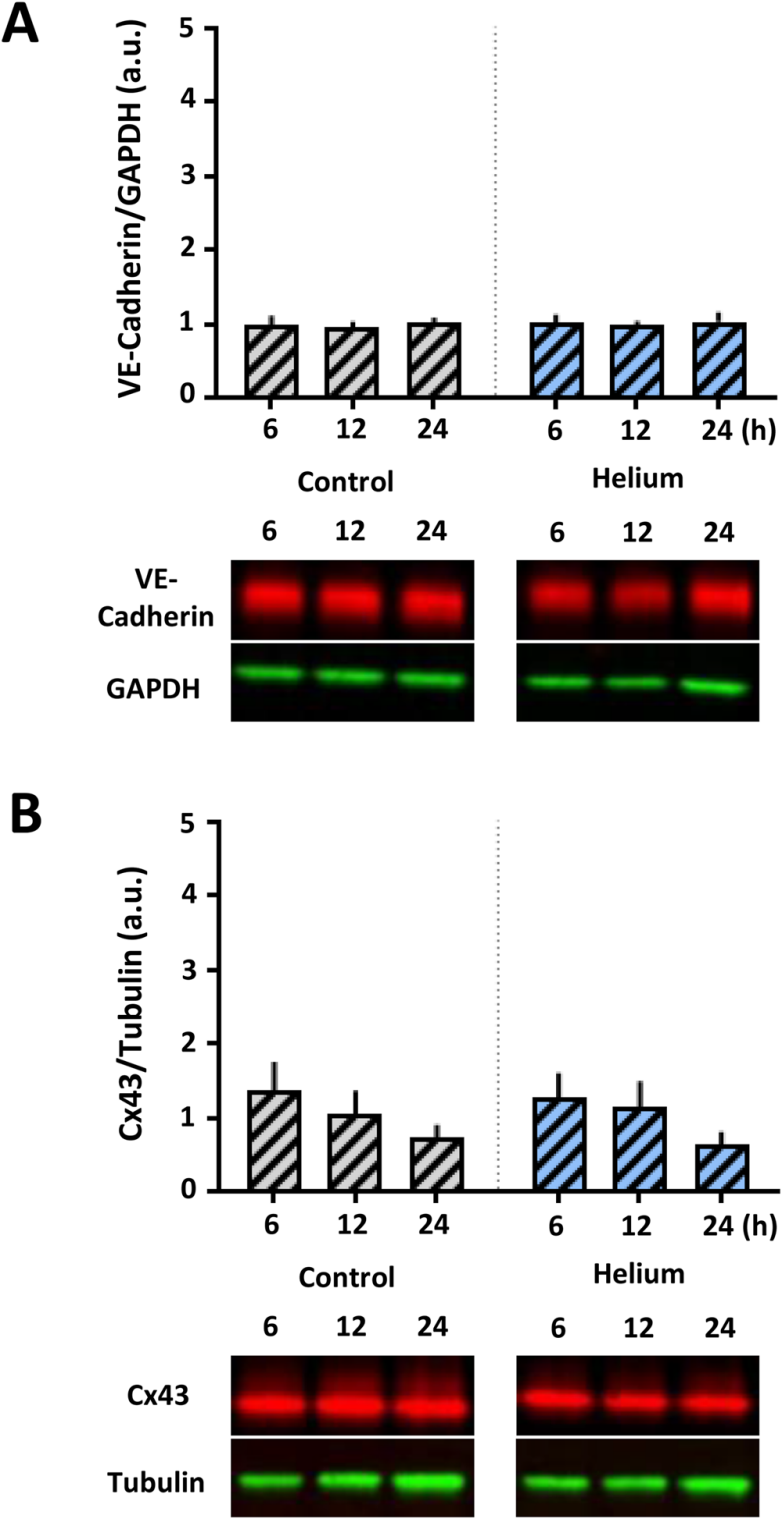
Effect of helium on VE-Cadherin and Cx43 in Cav-1 siRNA transfected HUVEC. Panel A: Western blot results of the ratio VE-Cadherin compared to GAPDH loading controls in Cav-1 siRNA transfected HUVEC at different time points following helium or control gas treatment. n = 7. Panel B: Western blot results of the ratio Cx43 compared to Tubulin loading controls in Cav-1 siRNA transfected HUVEC at different time points following helium or control gas treatment. n = 7. Cropped images of representative Western blot results are displayed below the respective graphs. Full-length blots are presented in Supplementary Figs S14–S15. Data are represented as mean ± SD, *p < 0.05. HUVEC = human umbilical vein endothelial cells.

Cav-1 knock down in HUVEC abolished helium induced permeability alterations and no significant differences between the helium and control group were seen (Fig. 5B). Furthermore, siRNA Cav-1 transfection abolished helium induced increase in VE-Cadherin and Cx43 (Fig. 6A,B). In HCAEC, helium did not significantly affect VE-Cadherin and Cx43 levels or the permeability, therefore Cav-1 siRNA transfection was not performed in HCAEC.

### Supernatant of helium treated cells influences permeability in HUVEC

Since helium leads to attenuated permeability and a translocation of Cav-1 to the supernatant, we investigated whether the Cav-1 enriched supernatant of helium treated HUVEC alone could mimic the effect of helium on HUVEC monolayer permeability.

**Figure 7:**
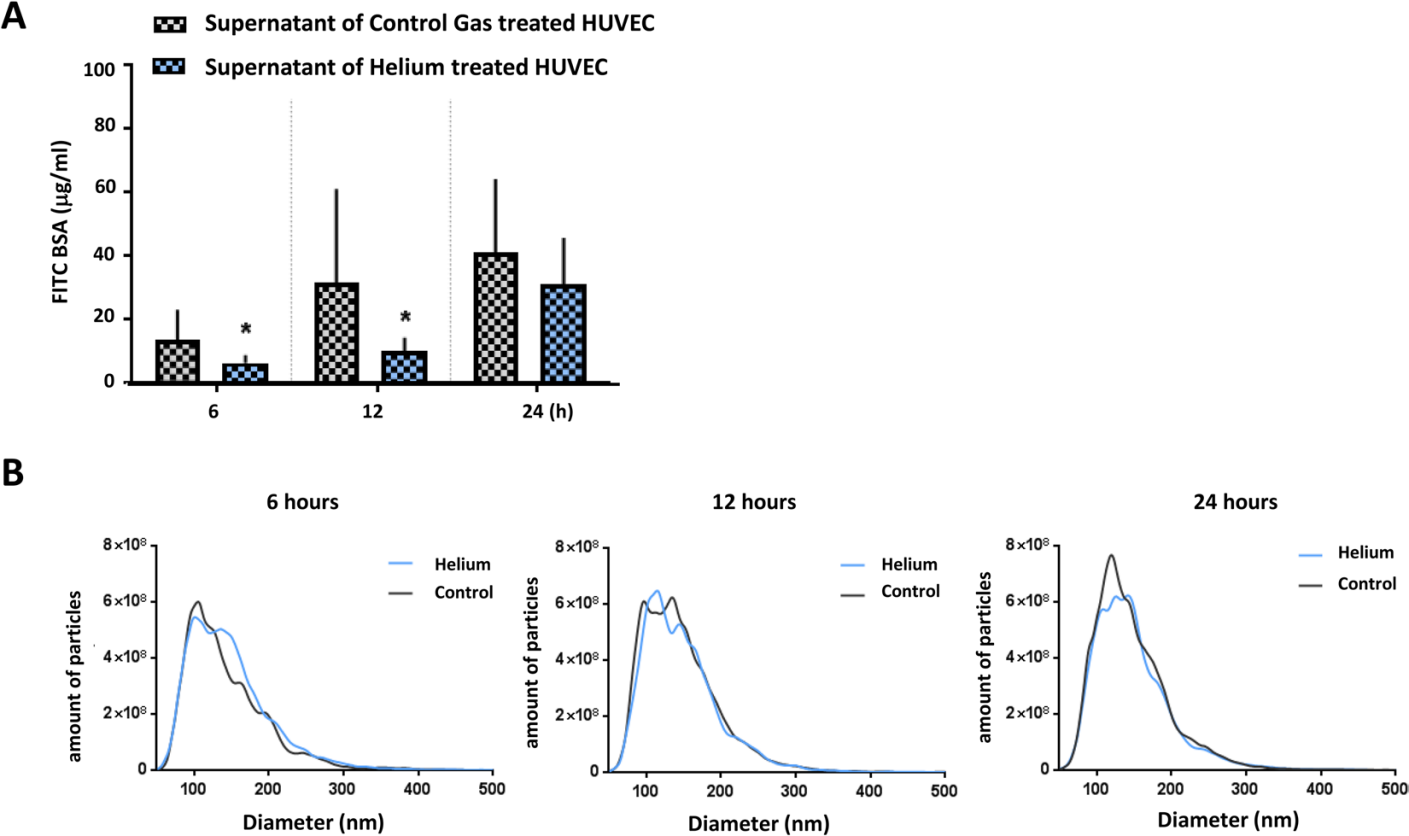
Supernatant analysis. Panel A: Effect of incubation with supernatant of helium and control gas treated cells on HUVEC Results of permeability of a confluent monolayer of HUVEC, estimated by the transfer of FITC-BSA, in HUVEC treated with supernatant of helium treated HUVEC at different time points. Controls were treated with supernatant of control gas treated HUVEC. n = 10. Panel B: Results of nanoparticle tracking analysis of supernatant after helium and control gas treated HUVEC. Results of nanoparticle tracking analysis of supernatant after helium and control gas treated HUVEC at different time points. n = 4. Data are represented as mean ± SD for panel A, and means for panel B, *p < 0.05. HUVEC = human umbilical vein endothelial cells.

Supernatant of HUVEC after helium treatment contains increased levels of Cav-1 (Fig. 2B). Supernatant of helium treated HUVEC was transferred to untreated, remote HUVEC. Supernatant obtained 6 and 12 hours after helium treatment (6.2 ± 2.5 and 9.9 ± 4.2, respectively) significantly decreased cellular permeability compared to supernatant obtained 6 and 12 hours after control gas treatment (13.3 ± 9.6 vs 31.3 ± 29.5, p < 0.05 respectively, Fig. 7A). Supernatant obtained at 24 hours did not affect permeability of HUVEC.

### Helium treatment alters particle formation in HUVEC

To obtain additional information about the vesicular components that are released by HUVEC after helium treatment, nanoparticle tracking analysis of HUVEC cell supernatants was performed. In the supernatant 6 hours after helium treatment an increased amount of particles was found compared to controls, see Fig. 7B. In the supernatant 12 and 24 hours after helium treatment this was abolished, Fig. 7B.

## Discussion

The major findings of this study are that exposure to the noble gas helium causes structural alterations to the cytoskeleton that preserve membrane stability and decrease vascular permeability in venous endothelial cells. This effect might be mediated by increased Cav-1 secretion to the supernatant at the expense of decreased cellular Cav-1 levels, and could be the mechanism behind helium induced organ protection.

Caveolin exists in 3 isoforms, each with different functions and origin. Cav-1 is ubiquitous in endothelial cells and was proven to have a signaling effect in remote preconditioning^24^.

For helium *post*conditioning, an increase of circulating Cav-3 was found, with increased levels of Cav-1 and -3 in the myocardial area-at-risk^25^. The findings of the present study match these earlier findings that helium increases circulating Cav-1 in the supernatant at the expense of decreasing cellular levels of Cav-1. This is in contrast to data from ischaemic and anaesthetic preconditioning, were increased cellular levels of Cav-3 were observed in cardiomyocytes^26^. Our data suggest that Cav-1 is involved in mediating the observed helium induced alterations in permeability, VE-Cadherin and Cx43, as Cav-1 knock down abrogated these effects. The essential difference between our study and previous preconditioning studies is that we did not use a damage model and purely investigated the effect of helium treatment in human cell lines.

Loss of cellular Caveolin and concomitant decrease in permeability has not been described before, yet the opposite is well known. In cancer research, increased Cav-1 was associated with increased vascular permeability affecting the blood-tumor barrier after exposure to various stimuli in brain microvascular endothelial cells and glioma cells^27^. Increased activated Caveolin resulted in increased vascular permeability in rats subjected to cardiopulmonary bypass^28^. The role of vascular permeability in preconditioning is unclear, although sevoflurane preconditioning protected endothelial cells by preserving VE-Cadherin levels and maintaining normal permeability^29,30^. Expression of VE-Cadherin is inversely related to permeability in endotoxin challenged lungs^31^. These findings match our results. We conclude that helium treatment increases the levels of VE-Cadherin and Cx43, leading to decreased vascular permeability.

Caveolae play a role in vascular permeability by mediating both, endocytosis and exocytosis^32^. Endothelial cells produce microvesicles via exocytosis, known as microparticles, exosomes, and apoptotic bodies. A novel concept in cancer research regards these apoptotic caspase 3 containing bodies as messengers warning nearby cells for loss of membrane integrity^33^. Larger extracellular vesicles and endothelial derived exosomes were shown to contain Cav-1^34^.

Prolonged exposure to helium increased microparticles production in HUVEC^35^. After TNF-α stimulation, helium further increased caspase-3 containing microparticles, possibly serving as signalosomes. Hypothetically, this increased microparticle formation could be mediated by Cav-1. In the present study, we showed that both, HUVEC and HCAEC increased levels of Cav-1 in the supernatant and this effect is likely due to Cav-1 mediated exocytosis. Furthermore, we showed that Cav-1 containing supernatant after helium treatment reduced permeability in untreated, remote HUVEC. This indicates that circulating Cav-1 particles are the mediating factor of these helium induced alterations of permeability. This was confirmed by abrogation of these effects in Cav-1 knock down cells, and concomitant absence of Cav-1 in the supernatant of these cells.

To further increase the hypothesis that helium increased microparticle formation via Cav-1, we performed nanoparticle tracking analysis and demonstrated increased amount of particles released 6 hours after treatment compared to controls. This effect was diminished after 12 hours and abolished at 24 hours. The timing of the increase matches our results of the Cav-1 levels in the supernatant suggesting these particles are indeed Cav-1 related. Previous research showed Caveolins produce vesicles ranging in size between 50–100 nm^36^. However, Cav-1 is able to oligomerizise^36^ and take-up various nanoparticles thereby increasing in size^37^ making it difficult to predict actual size. The observed population of particles in our study after helium treatment ranges between 120–180 nm and could possibly be Cav-1 related. Nanoparticle analysis ensures accurate measurement of particles from 50 nm, however cannot distinct between particles and vesicles^38^. Further analysis of the origin of the particles could be performed by electron microscopy.

Caveolin negatively regulates permeability, and in combination with a functional Cav-1 knock down by Cav-1 siRNA transfection this could theoretically lead to a double knock down of Cav-1. However, knocking down Cav-1 by transfection abolished the effects of helium treatment on permeability. This indicates that Cav-1 is involved in the pathway that mediates the effect of helium on the cytoskeleton, ultimately altering cellular permeability.

Supernatant of helium treated cells, containing Cav-1, is able to decrease permeability of remote HUVEC. This indicates that the essential step in helium conditioning might not be the decrease in cellular Cav-1 levels, but the rise of Cav-1 levels in the buoyant fraction, like the supernatant or the plasma.

Endothelial cells of different origin (arterial and vein) have different structure and functions; arteriolar cells mainly maintain vasomotor tone, while post capillary cells regulate leucocyte adhesion and trafficking^39^. This directly translates to observed differences in cytokine expression in HCAEC and HUVEC in quiescent state^40^, and exposed to shear stress^41^. Analysis in gene expression of arterial and venous cultured endothelial cells revealed marked differences in baseline gene expression; increased anti-inflammatory and oxidoreductase activity were found in venous endothelial cells^42^.

This might explain that in the present study different effects on F-actin localisation, cellular permeability, VE-Cadherin and Cx43 were observed in HCAEC after helium exposure. This was observed despite the fact that increased Cav-1 levels in the supernatant were found in both cell types. Even arterial cells can differ in their protein expression, as opposite results of VE-Cadherin expression were found in coronary artery cells and in aortic endothelial cells^43^.

This study is performed *in vitro*, and further research *in vivo* is needed before a possible translation of these data to a clinical setting.

Endothelial cells are sensitive to flow and shear stress, which is difficult to imitate *in vitro*. It is known that Caveolae control membrane tension and respond to flow, shear stress and stretch^32^. We cannot exclude that the absence of flow and shear stress influenced our results. The use of siRNA to accomplish a functional knock down of Cav-1 is often used and well described, however there are limitations to this technique.

First, siRNA is used for post-transcriptional gene silencing, creating a Cav-1 knock down model, but Cav-1 is not absent. In our study, 34.8% Cav-1 remained in the cell and could have influenced our results. It is of note that the used siRNA previously successfully specifically knocked down Cav-1^44^. However, we cannot exclude any off-target effects influencing our results.

In conclusion, exposure to helium decreases permeability in venous endothelial cells possibly by increasing VE-Cadherin and Cx43 levels, and this effect is mediated through a pathway involving Cav-1 *in vitro*. This mechanism could be responsible for the endothelial protection observed in helium preconditioning *in vivo*^1^. We postulate helium protects the endothelium by maintaining barrier function and preventing leakage and tissue edema, and ultimately preserving endothelial function.

## Material and Methods

**Figure 8:**
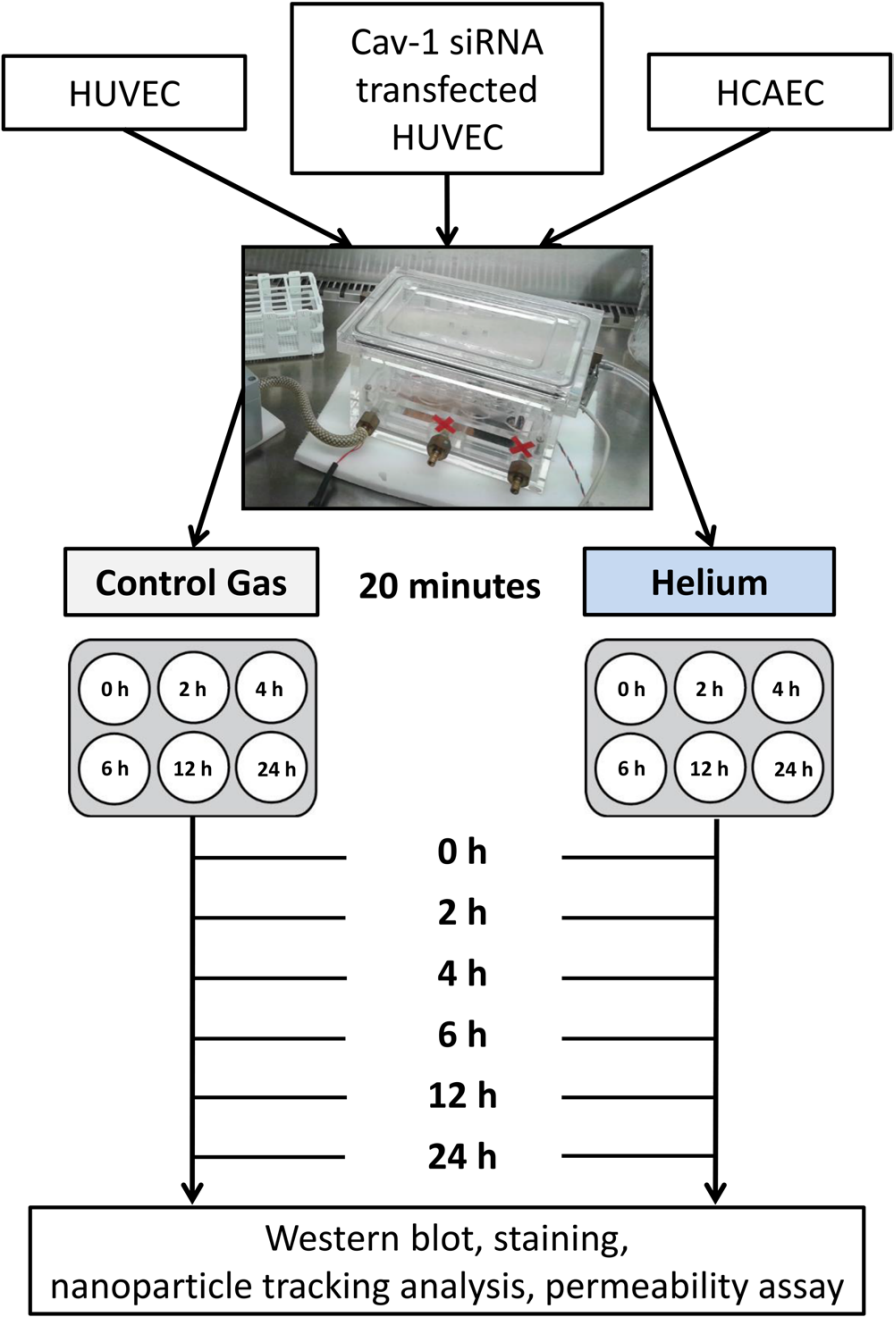
Protocol outline. Confluent cells (HUVEC, Cav-1 siRNA transfected HUVEC and HCAEC) were exposed to either helium (70%) or control gas for 20 minutes in a specialised temperature controlled gas chamber. Cells were harvested either directly after treatment or after 2, 4, 6, 12 and 24 hours respectively. HUVEC = human umbilical vein endothelial cells. HCAEC = human coronary artery endothelial cells.

For all experiments a specialised temperature controlled gas chamber (Fig. 8) was used. The different gas mixtures were administered via standard procedure as described before^45^, Using a gas analyzer (Capnomatic Ultima, Datex, Helsinki, Finland) the outlet gas concentrations were monitored throughout the experiment. The components of the mixture of helium were: 5% CO_2_, 25% O_2_, 70% He and for the mixture of control gas: 5% CO_2_, 25% O_2_, 70% N_2_. Both mixtures were provided by Linde Gas Benelux (Schiedam, The Netherlands).

### Materials

The materials used were from Sigma (Zwijndrecht, The Netherlands). Endothelial cell growth medium was obtained from Promocell (Heidelberg, Germany), medium M199 from PAN biotech (Aidenbach, Germany), Fetal Bovine Serum (FBS) from PAA (Colbe, Germany), Penicillin-Streptomycin, Amphotericin B, Trypsin-EDTA, L-glutamine from Gibco (Paisley, UK), and collagenase A from Roche (Mannheim, Germany). Sheep anti human von Willebrand factor-FITC from Serotec (Wiesbaden, Germany). HCAEC were from ATCC (Wesel, Germany).

### Isolation of human umbilical vein endothelial cells (HUVEC)

HUVEC were collected from human umbilical veins as described previously^45^. All experiments were performed in accordance with relevant guidelines and regulations of the AMC, Amsterdam (Statement of the Medical Ethics Review Committee AMC, Amsterdam, The Netherlands; Document Number W12–167#12.17.096). Moreover, all experiments were performed in accordance with the relevant standard operation procedures of the laboratory. HUVEC were identified using antibodies against von Willebrand factor. HCAEC were from ATCC (Wesel, Germany), and grown in vascular basal cell medium from ATCC (Wesel, Germany). Cells were cultured in gelatin (0.75%) coated flasks. Experiments were performed with HUVEC of passage 3 to 5, and HCAEC of passage 5 to 8.

### Experimental Protocol

The experimental protocol is outlined in Fig. 8. Before treatment with helium or control gas, the cells were washed three times with phosphate buffered saline (PBS) and fresh cell culture medium was added. Upon confluence, cells were exposed for 20 minutes to either helium (5% CO_2_, 25% O_2_, 70% He) or control gas (5% CO_2_, 25% O_2_, 70% N_2_) in a specialized temperature controlled gas chamber using a flow of 5 L/min. The gas temperature inside the gas chamber was kept at 37°C. Cells and supernatants were collected directly after treatment or after 2, 4, 6, 12 and 24 hours for Western blot analyses and immunofluorescence staining, and at 6, 12 and 24 hours for silver staining, nanoparticle tracking analysis and permeability measurements. To reduce risk of bias analyses were performed blinded.

### Immunofluorescence staining

Cells seeded on gelatin coated glass coverslips were fixed 0, 2, 4, 6, 12, and 24 hours after treatment using 4% formalin. After washing, the cells were permeabilized with 0.01% triton X-100 and blocked with 1% BSA. F-actin was stained overnight with Rhodamine-Phalloidine (Life Technologies, Eugene, OR, USA, 1:40) and Cav-1 with anti-Cav-1 (abcam, Cambridge, UK, 1:2000). Nuclei were stained with Hoechst and Cav-1 was detected using second antibody goat anti-rabbit DyLight 488 (abcam). The coverslips were mounted with Vecta Shield (Vector Laboratories, CA, USA) and screened using a Leica DMRA fluorescence microscope (Leica Microsystems, Wetzlar, Germany).

### Quantification of actin stress fibers

Six single cell images were taken per sample at 40x magnification to determine the proportion of the stress fibers of the border and the inner part within each cell. The fluorescent F-actin signal was analyzed using a MATLAB (Mathworks, Natick, MA, USA) script. To determine the proportion of the stress fibers, a ratio of the average intensity of the border and inner part of the cell was made, see Supplementary Material for details (Figure S1).

### Western blot analysis

Western Blotting was performed as described before^46^. After overnight incubation with primary antibodies: Cx43 (Cell Signaling Technology, Danvers, MA, USA, 1:1000); GAPDH (abcam, Cambridge, UK, 1:5000); VE-Cadherin (Cell Signaling Technology, Danvers, MA, USA, 1:1000); Alpha-Tubulin (Sigma-Aldrich, Zwijndrecht, The Netherlands, 1:10000); Cav-1 (abcam, Cambridge, UK, 1:10000), membranes were washed with phosphate buffered saline containing tween (PBS-T) and incubated with the appropriate secondary antibody (IRdye, Licor, Bad Homburg, Germany) for 1 hour at room temperature. After a final wash, membranes were scanned with the odyssey infrared imaging system (Licor, Bad Homburg, Germany) and quantification of the signals was performed with Odyssey Imaging Studio software (Licor, Bad Homburg, Germany).

### Nanoparticle tracking analysis

Size distribution and particle concentration in supernatant collected at different timepoints after treatment with helium or control gas was measured with the Nanosight (NS500, Malvern, Amesbury, UK) as described before^47^. Instruments were configured and calibrated with silica beads (100 nm diameter; Microspheres-Nanospheres, Cold Spring, NY, USA). Differed dilutions of fractions in PBS were made and 10 videos of a duration of 30 seconds were captured of each fraction. The threshold used to analyze all fractions was calculated by custom-made software (MATLAP v.7.9.0.529). The instrument software was employed to perform the analysis (NTA 2.3.0.15).

### Permeability assays

Cell permeability was determined by measuring the FITC-BSA concentration passing through a confluent cell monolayer on a polyester membrane. Cells were grown on 12-well plate transwell inserts (filter area 1.12 cm², pore diameter size 0.4 µm, Corning Costar, Cambridge, MA, USA) and upon confluency cells were treated with either helium or control gas according to protocol. FITC-BSA (10 mg/ml) was added to the wells and after incubation for 2 hours in the incubator, samples were collected from the lower compartment and stored at −80°C. Fluorescence was determined by a microplate fluorescence reader (BioTek, FLx800, Winooski, VT, USA) using KC4 software (BioTek).

### Transfection with siRNA for Cav-1

HUVEC were transfected at a confluency of 50–80% with siRNA for Cav-1 (Cav-1 sense 5′-CCCUAAACACCUCAACGAU-3′, Cav-1 antisense 5′-AUCGUUGAGGUGUUUAGGG-3′, Sigma-Aldrich, Zwijndrecht, The Netherlands) or negative control siRNA (Silencer Negative control siRNA, Ambion by Thermo Fischer Scientifics, Waltham, MA, USA) using Lipofectamine RNAiMax (Invitrogen by Thermo Fischer Scientifics, Waltham, MA, USA). SiRNA for Cav-1 or negative control siRNA was used at a final concentration of 100 nM and was left on the cells for 24 hours. After 72 hours, cells were lysed to determine protein knock down by Western blot analyses or experiments were started.

### Transfection with Block-iT Alexa Flour Red

HUVEC growing on glass cover slips at a confluency of 50–80% were transfected with the red fluorescent protein Block-iT Alexa Flour Red (Invitrogen by Thermo Fischer Scientifics, Waltham, MA, USA) with use of Lipofectamine RNAiMAX (Invitrogen by Thermo Fischer Scientifics, Waltham, MA, USA). Block-iT Alexa Flour Red was used at a final concentration of 100 nM. Negative control cells were treated with the same amount of Lipofectamine RNAiMAX, but without Block-iT Alexa Flour Red. Cells were fixed and stained with Hoechst nuclear staining 6, 12, 18 and 24 hours after adding Block-iT Alexa Flour Red and Lipofectamine RNAiMax or only Lipofectamine RNAiMAX to the cells. Images were taken using Leica DM-RA(X) Microscope (Leica Microsystems, Wetzlar, Germany).

### Statistics

Statistical analysis was performed using GraphPad Prism 7 (GraphPad Software, La Jolla, CA, USA). All data analysed for normality via frequency distribution graphs and the Pearson D’Agostino normality test, and were normally distributed. A student’s t-test was used to analyse the Cav-1 protein levels in Cav-1 siRNA transfected HUVECs and negative controls, and the permeability data of HUVEC treated with supernatant of helium treated cells. All remaining data were analysed using two-way analysis of variance (ANOVA) with Sidak correction for multiple testing. Values of p < 0.05 were considered statistically significant. All data are described as mean ± SD.

## Electronic supplementary material

Dataset 1
