# Supplementary material for: Helium alters the cytoskeleton and decreases permeability in endothelial cells cultured in vitro through a pathway involving Caveolin-1
Source: Sci Rep. 2018 Mar 19;8:4768. doi: 10.1038/s41598-018-23030-0 (PMC5859123; doi:10.1038/s41598-018-23030-0)
Supplement: Supplementary file 1 — Dataset 1 [file 41598_2018_23030_MOESM1_ESM.docx]

***Supplementary Information***

**Helium alters the cytoskeleton and decreases permeability in endothelial cells cultured in vitro through a pathway involving Caveolin-1**

**Authors** Kirsten F. Smit^1,+^

Moritz Konkel^1,2,+^

Raphaela Kerindongo^1^

Maximilian A. Landau^1,2^

Coert J. Zuurbier^1^

Markus W. Hollmann^1^

Benedikt Preckel^1^

Rienk Nieuwland^3^

Martin Albrecht^2^

Nina C. Weber^1,*^

^+^Both authors contributed equally to the present publication

**Affiliations** ^1^ Department of Anaesthesiology, Laboratory of Experimental Intensive Care and Anaesthesiology (L.E.I.C.A), Meibergdreef 9, 1100 DD Amsterdam, The Netherlands.

^2^Department of Anaesthesiology, UKSH, Campus Kiel, Germany.

^3^Laboratory of Experimental Clinical Chemistry, and Vesicle Observation Centre, Meibergdreef 9, 1100 DD Amsterdam, The Netherlands.

***Quantification of stress fibers***

Six single cell images were evaluated per sample at 40x magnification to determine the proportion of the stress fibers within each cell. The fluorescent F-actin signal was analyzed using a MATLAB (Mathworks, Natick, USA) script. First a binary mask of the foreground was created by thresholding the intensity value of the fluorescent-image using an unimodal background-symmetry method. A binary mask of the inner part of the cell was created by eroding the binary foreground mask with 10 pixels (≈1.9 µm) and a binary mask of the border by subtracting the inner part mask from the foreground mask. With these masks, an average intensity value per pixel of the fluorescence masked by the border and inner part was calculated. To determine the proportion of the stress fibers, a ratio was calculated of from the average intensity value of the border and inner part of the cell. The value 1 means the border and inner part have the same average intensity per pixel. So, there is no spatial distribution difference. If the value is >1 then the border contains more stress fibers and if value is < 1 the inner part contains more stress fibers. See figure S1.

**Figure S1: Quantification of stress fibers**

***Silver staining***

Supernatant collected at different time points after treatment with helium or control gas was analyzed by silver staining, as described before^44^. SDS polyacrylamide gel electrophoresis (PAGE) sample buffer (62.5 nM Tris-HCl, 2%SDS, 10%glycerol, 5%β-mercaptoethanol, all from Sigma) was added to the supernatants and subsequently they were boiled for 5 min. 10 μl of supernatant was separated by SDS-PAGE. The Silver Staining Kit, Protein plus one (GE Healthcare, Munich, Germany) was employed for silver staining.


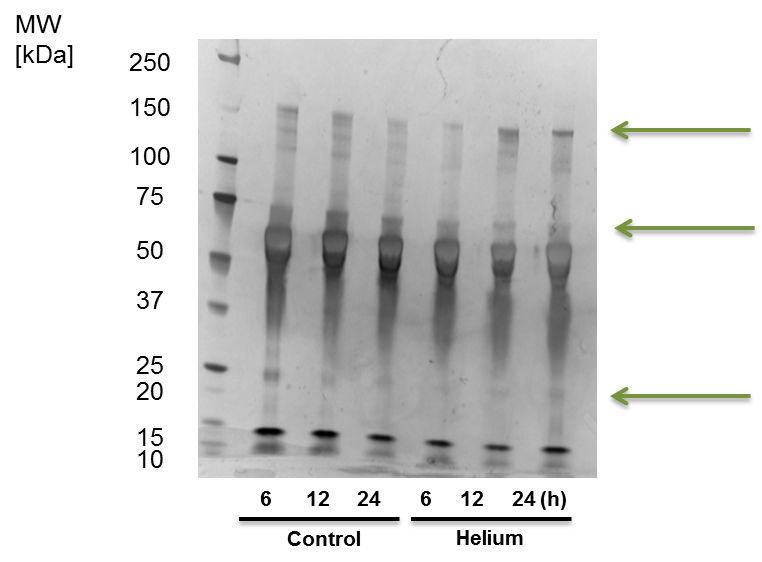


**Figure S2: Silver staining performed with HUVEC cell culture media derived 6, 12 and 24 hours after control gas or helium administration**

MW = molecular weight; kDa = kilo Dalton

In addition to the different amounts of released Cav-1 in the control and helium groups shown by WB, silver staining suggest a subtle differential release of several other proteins (25, 70 and 150 kDa, blue arrows below) between the control and helium treated cells. However, we do not know the nature of the respective proteins yet.

***Supplementary figures***

**Figure S3: Effect of helium on permeability in HCAEC**

Results of helium on permeability of a confluent monolayer of HCAEC, estimated by the transfer of FITC-BSA, at different time points compared to control gas. n=6.

Data are represented as mean±SD, HCAEC = human coronary artery endothelial cells.

**Figure S4: Transfection with Lipofectamine RNAiMax**

A negative control for the transfection with a red fluorescent protein (figure 5B). Immunofluorescent staining of HUVEC after treatment with Lipofectamine RNAiMax without a red florescent protein. Cells were fixed 24 hours after transfection.

***Full-length blots***

**Figure S5: Full-length blot of Figure 2A**

Panel A: Cav-1.

Panel B: GAPDH.

Areas marked with white dashed rectangles are included in Figure 2A. Left: control gas treatment, right: helium treatment.

**Figure S6: Full-length blot of Figure 2B**

Panel A: Cav-1.

Panel B: Albumin.

Areas marked with white dashed rectangles are included in Figure 2B. Left: helium treatment, right: control gas treatment.

**Figure S7: Full-length blot of Figure 2C**

Panel A: Cav-1.

Panel B: GAPDH.

Areas marked with white dashed rectangles are included in Figure 2C. Left: control gas treatment, right: helium treatment.

**Figure S8: Full-length blot of Figure 2D**

Panel A: Cav-1.

Panel B: Albumin.

Areas marked with white dashed rectangles are included in Figure 2D. Left: control gas treatment, right: helium treatment.

**Figure S9: Full-length blot of Figure 3A**

Panel A: VE-Cadherin.

Panel B: GAPDH.

Areas marked with white dashed rectangles are included in Figure 3A. Left: control gas treatment, right: helium treatment.

**Figure S10: Full-length blot of Figure 3B**

Panel A: Cx43.

Panel B: Tubulin.

Areas marked with white dashed rectangles are included in Figure 3B. Left: control gas treatment, right: helium treatment.

**Figure S11: Full-length blot of Figure 3C**

Panel A: VE-Cadherin.

Panel B: GAPDH.

Areas marked with white dashed rectangles are included in Figure 3C. Left: control gas treatment, right: helium treatment.

**Figure S12: Full-length blot of Figure 3D**

Panel A: Cx43.

Panel B: GAPDH.

Areas marked with white dashed rectangles are included in Figure 3D. Left: control gas treatment, right: helium treatment.

**Figure S13: Full-length blot of Figure 4A**

Panel A: Cav-1.

Panel B: GAPDH.

Areas marked with white dashed rectangles are included in Figure 4A. Left: transfection with siRNA for Cav-1, right: transfection with negative control siRNA.

**Figure S14: Full-length blot of Figure 6A**

Panel A: VE-Cadherin.

Panel B: GAPDH.

Areas marked with white dashed rectangles are included in Figure 6A. Left: control gas treatment, right: helium treatment.

**Figure S15: Full-length blot of Figure 6B**

Panel A: Cx43.

Panel B: Tubulin.

Areas marked with white dashed rectangles are included in Figure 6B. Left: control gas treatment, right: helium treatment.
